# Multivariate Cluster-Based Multifactor Dimensionality Reduction to Identify Genetic Interactions for Multiple Quantitative Phenotypes

**DOI:** 10.1155/2019/4578983

**Published:** 2019-07-11

**Authors:** Hyein Kim, Hoe-Bin Jeong, Hye-Young Jung, Taesung Park, Mira Park

**Affiliations:** ^1^Department of Statistics, Korea University, Seoul,02841, Republic of Korea; ^2^Department of Statistics, Seoul National University, Seoul, 08826, Republic of Korea; ^3^Department of Preventive Medicine, Eulji University, Daejeon, 34824, Republic of Korea

## Abstract

To understand the pathophysiology of complex diseases, including hypertension, diabetes, and autism, deleterious phenotypes are unlikely due to the effects of single genes, but rather, gene-gene interactions (GGIs), which are widely analyzed by multifactor dimensionality reduction (MDR). Early MDR methods mainly focused on binary traits. More recently, several extensions of MDR have been developed for analyzing various traits such as quantitative traits and survival times. Newer technologies, such as genome-wide association studies (GWAS), have now been developed for assessing multiple traits, to simultaneously identify genetic variants associated with various pathological phenotypes. It has also been well demonstrated that analyzing multiple traits has several advantages over single trait analysis. While there remains a need to find GGIs for multiple traits, such studies have become more difficult, due to a lack of novel methods and software. Herein, we propose a novel multi-CMDR method, by combining fuzzy clustering and MDR, to find GGIs for multiple traits. Multi-CMDR showed similar power to existing methods, when phenotypes followed bivariate normal distributions, and showed better power than others for skewed distributions. The validity of multi-CMDR was confirmed by analyzing real-life Korean GWAS data.

## 1. Introduction

In genome-wide association studies (GWAS), genotype data from a large number of single nucleotide polymorphisms (SNPs) are collected, to associate SNPs with traits of interest [1]. Not only single gene effects, but also interaction effects, between genes, play important roles in complex diseases such as hypertension, diabetes, and autism. By identifying gene-gene interactions (GGIs), we expect to increase statistical power, to detect associations. Moreover, we also hope to clarify the biological pathways underlying human diseases, by detecting interactions between loci [2].

In many cases, a phenotype is considered, and there are various studies on statistical methods for finding GGIs, for univariate phenotypes. For studying qualitative traits, as in the case-control studies, one simple way for identifying genetic interaction is to fit a logistic regression model (LRM) that includes main effects and relevant interaction terms. However, LRMs perform poorly when there is a dimensionality problem. Another well-known approach is a multifactor dimensionality reduction (MDR) method [3, 4], which reduces dimensions by converting a high-dimensional to a one-dimensional model. The genotype combinations are classified as either “high-risk” or “low-risk,” depending on the ratio of cases to controls, for each genotype combination. Thus, an MDR can avoid the issues of sparse data cells and overparameterization of models [2] and can outperform LRMs, for detecting higher order GGIs [5]. Recently, various approaches such as using multiple contingency table (MODENDR) [6] or particle swarm optimization method (PBMDR) have been developed [7].

Due to its superior performance there are now various extensions of MDR, including ordinal phenotypes, quantitative phenotypes, survival information, and odds-ratio-based analysis [8–11]. One specific extension of MDR, generalized MDR, which is applicable to both dichotomous and continuous traits, was proposed [12]. However, GMDR does not provide a computationally efficient algorithm that is easy to implement, and it still requires a dichotomous outcome in the data file [9]. As an alternative, quantitative MDR (QMDR) modified MDR's constructive induction algorithm, which assigns a genotype to either the high- or low-risk groups by comparing the local and global means and then applies a* t*-test to compare the means of the two groups. More recently, cluster-based MDR (CL-MDR), which is less sensitive to outliers and distributional assumptions, was also developed [13, 14]. Compared to QMDR, CL-MDR was shown to yield higher power, when the phenotype distribution is skewed. However, CL-MDR was developed only for univariate phenotype rather than multivariate phenotypes.

When considering multiple phenotypes, it becomes more difficult to find GGIs. Thus, most GWAS studies still focus on one trait to identify genetic variants associated with common complex traits, even though multiple phenotypes or repeated measurements of phenotypes are available. However, in the study of a complex disease, several correlated traits are often measured at the same time as risk factors for the disease. For example, it is known that intermediately correlated phenotypes, such as Factors VII, VIII, IX, XI, and XII and von Willebrand factor, jointly predict the risk of developing thrombosis [1, 9, 20]. By modeling multivariate disease-related traits, the power to detect associations between genes and diseases is expected to increase. Analyses of multiple traits have been successful in analyzing various complex diseases. In general, the multivariate approach has several advantages over the univariate approach considering one trait at a time. For example, the multivariate approach can consider several traits simultaneously in one model and hence it can take into account the correlation among traits. As a result, the multivariate approach would have higher power to detect pleiotropic genes and it can identify genetic variants not easily detected by the univariate approach [21].

There is relatively less GGI research on multivariate traits case. To deal with multiple phenotypes, generalized estimating equations (GEE)-GMDR is an extension of GMDR method, using the GEE model [22]. Multi-QMDR, which extends QMDR to multivariate cases, has also been proposed [5]. Multi-QMDR classifies samples into high- vs. low-risk groups, by using summary statistics, based mainly on principal component scores. After classification, the two groups' mean vectors are compared, using Hotelling's *T*^2^ statistic. While this approach is simple and intuitive, it is not appropriate when the distribution of phenotypes is not symmetric and/or skewed and is also sensitive to outliers.

Recently, several MDR extensions were proposed using the fuzzy set theory [23–27]. Such fuzzy set-based MDR methods classify high-risk or low-risk groups as equivalent to defining the degree of membership in high- and low-risk groups. By adopting the fuzzy set theory, fuzzy set-based MDR methods take into account the uncertainty of this binary classification. Fuzzy set-based MDR methods allow the possibility of partial membership into high- and low-risk groups, through a membership function, which transforms the degree of uncertainty into a [0,1] scale. Then, the best genotype combinations can be selected, maximizing a new fuzzy set-based accuracy measure. Specifically, fuzzy MDR [23] was proposed to detect GGIs for a binary trait and was shown to yield higher power than the original MDR. Furthermore, an empirical fuzzy MDR (EF-MDR) model [24] was proposed to overcome the selection problem of tuning parameters in the original fuzzy MDR, while a fuzzy set-based generalized multifactor dimensionality reduction (FGMDR) model [25] was also proposed for covariate adjustment, for both quantitative and binary traits. More recently, a faster version of EFMDR was developed [26]. Fuzzy C-means-based entropy approach [27] was proposed as the method to detect GGIs for binary trait. It uses two measures: correct classification rate (FCMEMDR-CCR) and likelihood ratio (FCMEMDR-LR).

Here, we propose a new method to detect GGIs for multiple quantitative traits. The main idea of our method to detect GGIs for multiple quantitative traits lies in combining fuzzy clustering with a modified multifactor dimensionality reduction (MDR) approach, named “multivariate cluster MDR” (multi-CMDR). Like other MDR-based methods, multi-CMDR also pools multiple genotype combinations into two groups and uses them as a new attribute, reducing multidimensional space into one dimension. To classify genotype combinations, we first performed fuzzy k-means clustering and computed a threshold, representing the ratio of the sum of the membership degrees of the two groups. Each multilocus genotype is labeled by comparing the local ratio, in each multilocus genotype, to the global ratio. Then, multi-CMDR identifies the best genotype model, using Hotelling's *T*^2^ statistic. To find the overall best model, 10-fold cross-validation (CV) is performed and the best model is chosen which has the largest CV consistency. Unlike other GGI methods for multiple quantitative traits, multi-CMDR is robust to outliers and underlying distributions.

We first introduce the multi-CMDR method in detail in Section 2. We next present a simulation study in Section 3, to show the performance of the proposed methods by comparing them to other methods, such as multi-QMDR. For a phenotype distribution, multivariate normal and multivariate gamma distributions are considered. In Section 4, we apply our method to three lipid-related phenotypes data extracted from the GWA study of the Korean Association Resource (KARE) project, as an illustration. We end with some conclusions in Section 5.

## 2. Materials and Methods

**Figure 1:**
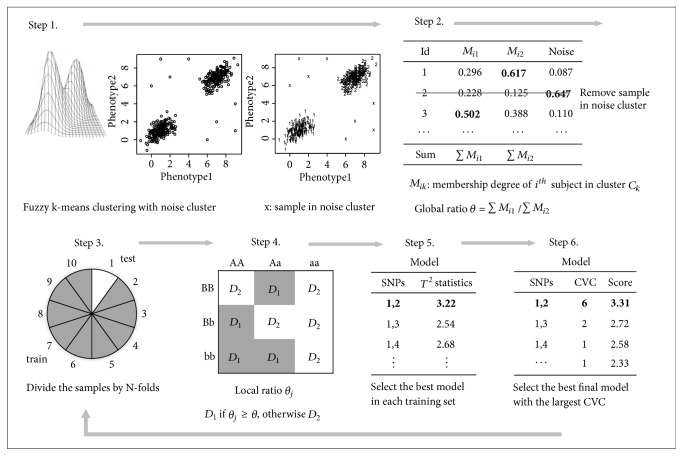
Summary of the multi-CMDR algorithm in the case of 10-fold and 2^nd^-order gene-gene interactions.

In this section, we introduce a new procedure, multi-CMDR, for finding GGIs for multiple continuous phenotypes. Similar to other MDR-based methods, multi-CMDR pools multiple genotype combinations into two groups and uses them as a new attribute that reduces a multidimensional space into only one dimension. The detailed algorithm is described in Figure 1 and the multi-CMDR pseudocode is presented in Pseudocode 1.

*Step 0. *Preprocessing.

- Suppose there are *n* samples, with *p* SNP data points and *q* continuous phenotypes. Let *Y*_*i*_ = (*y*_*i*1_, *y*_*i*2_, ⋯, *y*_*iq*_)^*T*^ be the phenotype vector and let *X*_*i*_ = (*x*_*i*1_, *x*_*i*2_, ⋯, *x*_*ip*_)^*T*^ be the genotype vector for the *i*-th subject, respectively, (*i* = 1, ⋯, *n*).
- Standardize all the phenotypes to have a mean of zero and no unit variance.

*Step 1*. Perform fuzzy k-means clustering.

(i) Perform fuzzy k−means clustering with *k* = 2 using phenotype information. Here, we make an additional pseudocluster (i.e., “noise cluster”) during the process of clustering [28]. Samples are then allocated into one of three clusters: two good cluster groups and one noise cluster. In this study, we set the noise cluster threshold value to equal the average squared Euclidean distance between samples. Two good clusters and one noise cluster are obtained by minimizing the following *J*_*Noise*_:
  (1) $$\begin{array}{c} J_{Noise}=\overset{n}{\underset{i=1}{\sum }}\;\overset{2}{\underset{k=1}{\sum }}{M_{ik}}^{m}{\left(\;x_{i}-c_{k}\;\right)}^{2}+\overset{n}{\underset{i=1}{\sum }}\delta^{2}{\left(\;1-\sum_{k=1}^{2}M_{ik}\;\right)}^{m} \end{array}$$
such that *M*_*ik*_ ∈ [0,1], ∑_*k*=1_^3^*M*_*ik*_ = 1. *M*_*ik*_ is the membership degree of the *i*^*th*^ subject in group *C*_*k*_, *c*_*k*_ is the center of the cluster *C*_*k*_, *M*_*i*3_ is the membership degree of the noise cluster, *m*(>1) is the fuzzifier parameter which defines the group's fuzziness (usually *m* = 2), and *δ* is a squared distance of each data point to the noise cluster.

*Step 2*. Trim the data and calculate the global ratio.

(i) Data are trimmed by removing all the samples in the noise cluster. The remaining samples have membership degrees for each of the two groups. Denote these two groups as *C*_1_ and *C*_2_. The membership degree of the *i*^*th*^ subject in group *C*_*k*_  (*k* = 1,2) is given by
  (2) $$\begin{array}{c} M_{ik}=\frac{1}{\sum_{j=1}^{2}{\left[{\left(x_{i}-c_{k}\right)}^{2}/{\left(x_{j}-c_{j}\right)}^{2}\right]}^{1/\left(m-1\right)}} \end{array}$$
(ii) Calculate global ratio *θ*:
  (3) $$\begin{array}{c} \theta =\frac{\sum_{i=1}^{n}M_{i1}}{\sum_{i=1}^{n}M_{i2}}, \end{array}$$
where *M*_*ik*_ is the membership degree of the *i*^*th*^ subject in cluster *C*_*k*_.

*Step 3*. Divide the samples by N-folds.

- For N-folds, split the cross-validation (CV) samples randomly into N subgroups of equal size. Let N-1 sets of samples be the training dataset and let the remaining dataset be the test dataset used for evaluating the model.

*Step 4*. Calculate the local ratio.

(i) To find the *m*^*th*^-order gene-gene interactions, select a set of m SNPs from a pool of SNPs. Calculate the local ratio *θ*_*j*_ for the *j*^*th*^ genotype combination in the training set. *θ*_*j*_ is the ratio of the sum of membership degrees of the samples belonging to *C*_1_ to that belonging to *C*_2_:
  (4) $$\begin{array}{c} \theta_{j}=\frac{\sum_{i=1}^{n_{j}}M_{ij1}}{\sum_{i=1}^{n_{j}}M_{ij2}},\;\left(j=1,\cdots ,3^{m}\right) \end{array}$$
where *M*_*ijk*_ is the membership degree of the *i*^*th*^ subject with the *j*^*th*^ genotype combination, in cluster *C*_*k*_.
(ii) Label each genotype combination either “*D*_1_,” if *θ*_*j*_ ≥ *θ*, or “*D*_2_,” if *θ*_*j*_ < *θ*.

*Step 5*. Calculate the test statistic.

(i) Calculate Hotelling's *T*^2^ statistic, for both training and testing datasets, to test differences of the mean vectors between the *D*_1_ and *D*_2_ groups:
  (5) $$\begin{array}{c} T^{2}={\left(\;{\overset{-}{x}}_{1}-{\overset{-}{x}}_{2}\;\right)}^{T}{\left[\;\left(\;\frac{1}{n_{1}}+\frac{1}{n_{2}}\;\right)S_{pooled}\;\right]}^{-1}\left(\;{\overset{-}{x}}_{1}-{\overset{-}{x}}_{2}\;\right) \end{array}$$

 where *n*_1_ is the number of observations in group *D*_1_ and *n*_2_ is the number of observations in group *D*_2_; *x*_1*i*_ is *i*^th^ observation of *D*_1_; *x*_2*j*_ is *j*^th^ observation of *D*_2_.

(6) x−1=1n1∑i=1n1x1i,x−2=1n2∑j=1n2x2j,S1=1n1−1∑i=1n1x1i−x−1x1i−x−1T,S2=1n2−1∑j=1n2x2j−x−2x2j−x−2T,Spooled=n1−1n1+n2−2S1+n2−1n1+n2−2S2

(ii) The model with the largest statistic in the training data is chosen as the best model. Statistics for the test data will be performed later.

*Step 6.* Find the final best model and obtain the empirical p-value.

(i) Repeat Steps 4 and 5 N times, for each fold, and count the number of specific SNP combinations for the best model. We call this cross-validation consistency (CVC).
(ii) Find the best final interaction model, i.e., the one with the largest CVC.
(iii) Derive the final statistic for the best model by averaging N *T*^2^ statistics for the test data and let this statistic be *T*^2^_*test*_.
(iv) To evaluate the statistical significance of the best model, perform a permutation test and obtain the empirical p-value. Generate *B* permuted datasets by shuffling only the phenotype vector *Y*_*i*_ across individuals while fixing the genotype vector *X*_*i*_. This way of shuffling nullifies the association between the phenotype and genotype vectors, while preserving the correlation structures within their components. Perform the multi-CMDR and calculate *T*^2^ statistics for each permuted dataset. *B* test statistics are in *T*^2^_*null*_. The empirical p-value is calculated as
  (7) $$\begin{array}{c} p-value=\frac{1}{B}\overset{B}{\underset{i=1}{\sum }}I\left(\;{T^{2}}_{null}>{T^{2}}_{test}\;\right) \end{array}$$
where *I*(*x*) is indicator function, returning 1 if *x* is true, otherwise 0.

## 3. Results and Discussion

### 3.1. Simulation Analysis

In this section, we conducted simulations to compare the performance of the proposed multi-CMDR method, with multi-QMDR and univariate QMDR methods. We also compared the performance of the two versions of multi-CMDR. One version is a nontrimmed version of multi-CMDR. That is, the noise cluster is not generated in the fuzzy clustering step. The other version uses k-means clustering, without considering membership score. For multi-QMDR methods, the First Principal Component (FPC) was used to classify each cell into high- or low-risk groups, as previously described [5]. For a univariate approach, QMDR was performed for each phenotype, separately. All of these methods were compared in terms of their hit-ratios, representing the ratio at which the true causal SNP pair is identified by the best model.

We then generated a multivariate normal distribution and a multivariate gamma distribution for phenotypes. We used 70 different penetrance functions that define a probabilistic relationship with disease-causal interaction. The models consisted of 7 different heritability values (0.01, 0.025, 0.05, 0.1, 0.2, 0.3, and 0.4) and 2 different minor allele frequencies (MAFs, 0.2 and 0.4). A total of 5 models for each of the 14 heritability-minor allele frequency combinations were considered. Thus, a total of 70 models were generated. The details of the 70 penetrance functions are given in [29]. For every 70 models, 100 datasets were generated. For each dataset, the sample size was 400, and we considered 20 SNPs and 2 continuous phenotypes. SNP1 and SNP2 denoted disease-causal SNP interactions. We used 10-fold cross-validation to determine best overall model.

#### 3.1.1. Multivariate Normal Distribution

For the multivariate normal distributed case, two continuous phenotype values, *Y* = (*Y*_1_, *Y*_2_)^*T*^, were associated with SNP_1_ and SNP_2_, respectively, and were generated from the bivariate normal distribution,

(8) $$\begin{array}{c} Y\;|\;\left(\;SNP_{1}=i,SNP_{2}=j\;\right)\sim MN\left(\;\mu_{ij},\Sigma \;\right), \end{array}$$

where $\mu_{ij}=\left(\begin{array}{c} f_{ij}\\ f_{ij} \end{array}\right)$ and $\Sigma =\left(\begin{array}{cc} 1 & \rho \\ \rho & 1 \end{array}\right)$, and *f*_*ij*_ is the element from the *i*^*th*^ row and *j*^*th*^ column of a penetrance function, representing the two functional interacting SNPs. From this, we considered 3 different *ρ*  s  :  *ρ* = 0, 0.25, 0.5. We used R software to generate simulation data. For multivariate normal distributed cases, we used mvrnorm() function in MASS package in R.

**Figure 2:**
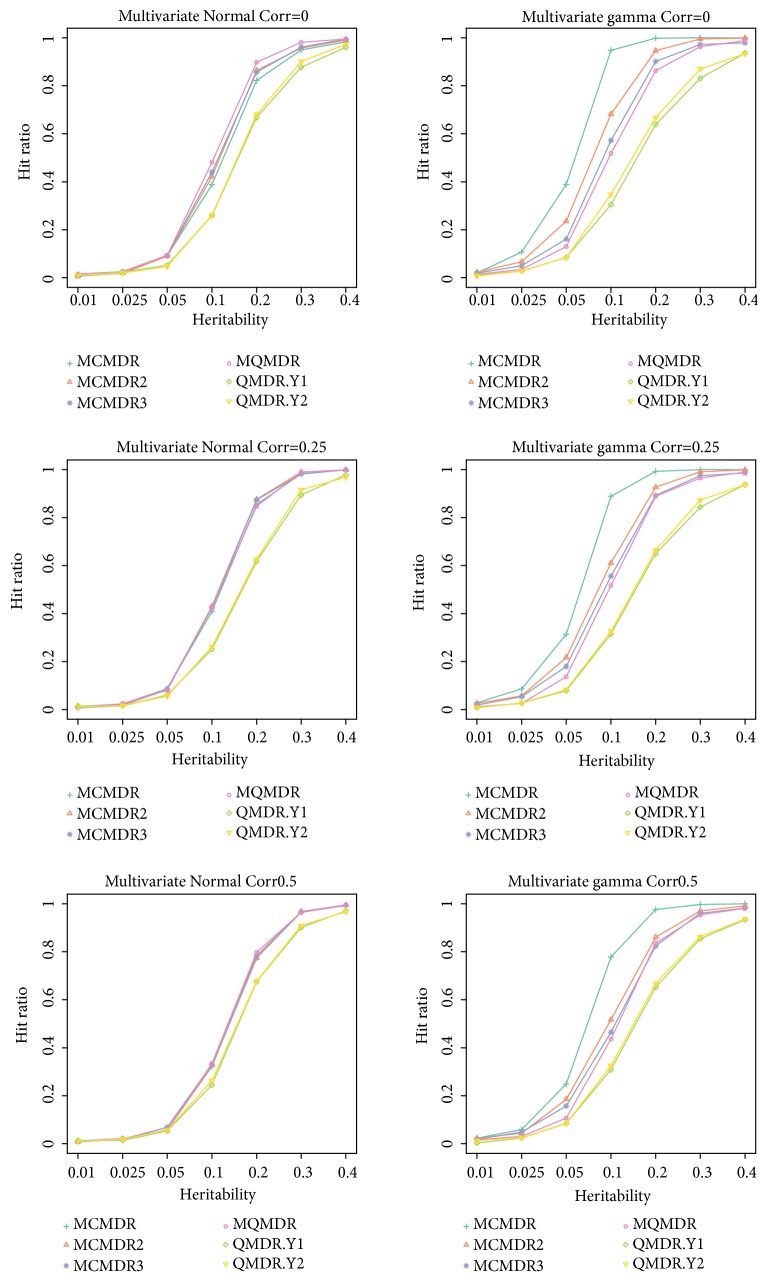
Hit-ratios for a multivariate normal distribution and multivariate gamma distribution. MCMDR (multi-CMDR), MCMDR2 (multi-CMDR without trimming, MCMDR3 (multi-CMDR, without membership score), MQMDR (multi-QMDR), QMDR.Y1 (QMDR with *Y*_1_), and QMDR.Y2 (QMDR with *Y*_2_).

The hit-ratios for each heritability values are reported in Figure 2. In the bivariate normal distribution case, all the multivariate methods were generally more powerful than the univariate QMDR methods. As the correlation increased, however, the difference between multivariate and univariate methods decreased. All multivariate methods showed similar performance. In the case of zero correlation, multi-QMDR showed slightly better performance than multi-CMDR. The hit-ratios of multi-CMDR, with trimming, were similar to those of multi-CMDR without trimming. That is, there was no effect of trimming outliers in multi-CMDR for the bivariate normal distribution case. The lower the correlation, the higher the hit-ratio, when the values of heritability were 0.05, 0.1, and 0.2. This is because the lower the correlation, the more unique information for each variable. In a similar context, when the correlation was high, the hit-ratios of the multivariate and univariate methods were similar.

#### 3.1.2. Multivariate Gamma Distribution

For the skewed distribution, we generated bivariate gamma distribution using Gaussian copula [30]. In the Gaussian copula, the correlation matrix is responsible for the dependence. We used the same correlation structure, for the bivariate normal case. When the marginal distributions were continuous, a bivariate distribution could be defined by the density of the following form:

(9) $$\begin{array}{c} g\left(\;y_{1},y_{2};\Sigma \;\right)=c\left(\;u,\Sigma \;\right)f_{1}\left(\;y_{1}\;\right)f_{2}\left(\;y_{2}\;\right), \end{array}$$

where *c*(*u*, Σ) represents the copula density, $\Sigma =\left(\begin{array}{cc} 1 & \rho \\ \rho & 1 \end{array}\right)$, *f*_1_,*f*_2_ are marginal probability density functions, and *g* is joint density function. The Gaussian copula density is then defined as follows:

(10) $$\begin{array}{c} c\left(\;u,R\;\right)={\left|\;\Sigma \;\right|}^{1/2}exp\left[\;-\frac{{\tilde{u}}^{T}\left(\Sigma^{-1}-I\right)\tilde{u}}{2}\;\right] \end{array}$$

where $\tilde{u}=(\Phi^{-1}(u_{1}),\Phi^{-1}(u_{2}){)}^{T}$, *u*_*i*_ = *F*_*i*_(*y*_*i*_), *i* = 1,2, and Φ^−1^ is the inverse cumulative distribution function of the standard normal distribution; *F*_1_, *F*_2_ are marginal cumulative distribution functions. The forms of two gamma distributions, *f*_1_(*y*_1_) and *f*_2_(*y*_2_), are as follows:

(11) Y1 ∣ SNP1=i,SNP2=j~Gammafij2,1fij,Y2 ∣ SNP1=i,SNP2=j~Gammafij2,1fij

From this, we considered 3 different *ρ* s  :  *ρ* = 0, 0.25, 0.5. For multivariate gamma distributed cases, we used mvdc(),normalCopula(),rMvdc() functions in copula package in R.

In Figure 2, we observed that the proposed multi-CMDR outperformed the QMDR and the multi-QMDR, for all ranges of heritability, for the bivariate gamma distribution case. Also, multi-CMDR, without trimming, performed better than multi-QMDR. For the bivariate gamma distribution, the lower the correlation, the higher the overall hit ratio. The difference of hit-ratios between multi-CMDR and other methods was greatest when the heritability was 0.1. As the correlation increases, the differences between hit ratios of the multivariate methods, except multi-CMDR, decrease.

To sum up, the power of proposed multi-CMDR is similar to that of multi-QMDR, for symmetric distribution while it outperformed multi-QMDR for the skewed distribution. Moreover, the powers of the two different versions of multi-CMDR were also slightly better than those of multi-QMDR, in skewed phenotype distributions. For all situations, multivariate methods performed better than univariate methods. Results for each combination of two minor allele frequency (MAF) values and 5 models are presented in the supplemental materials (Supplemental Figures 1-6).

#### 3.1.3. Empirical False Positive Rate

We computed empirical false positive rate. To compute empirical false positive rate, we permuted phenotypes over individuals for each case to generate null data. The selection rate of each SNP pair in null data is $1/\left(\begin{array}{c} 20\\ 2 \end{array}\right)$= 0.0053. To compute empirical false positive rate, we counted the number of detecting a specific SNP combination, SNP1 and SNP2, as the best model. Overall, empirical false positive rates of each method are closed to the expected value 0.0053. Results for empirical false positive rates of each method are presented in the supplemental materials (Supplemental Tables 1-6).

### 3.2. Real Biological Data Analysis

**Figure 3:**
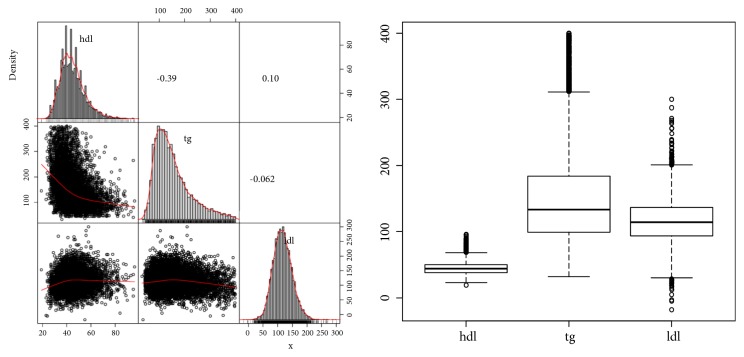
(Left) Scatter plots, histograms, and correlations between phenotypes. (Right) Box plots of phenotypes.

For real-life data analysis, three lipid-related phenotypes' data, retrieved from the Korean Association Resource (KARE) project [31], were considered to evaluate the proposed multi-CMDR. Three lipid-related phenotypes consisted of high-density lipoprotein cholesterol (HDL), low-density lipoprotein cholesterol (LDL), and triglyceride (TG). After removing those observations with at least one missing phenotype value, there were 8,581 samples remaining. The largest absolute value of correlation between three phenotypes was 0.39 (Figure 3). Among 344,596 SNPs, we used 324 SNPs selected in [5] for this analysis.

We then applied the proposed multi-CMDR to search for the best second interaction model, again by using 10-fold CV.  Table 1 displays the best 1^*st*^ and 2^*nd*^-order SNP combinations, identified by the proposed multi-CMDR. In addition to the best model, which has the highest CVC, Table 1 shows other candidate models selected from the best models, in every 10 training datasets. To see if these SNP combinations have been previously detected, one previous study [5] reported the best SNP combinations found in this study, including those described in Table 1.

For 1^*st*^-order analysis, rs1106280 was selected as the best model with the highest CVC. rs11066280 was identified as significantly associated with metabolism, TGs, and HDLs [5, 15] and was selected as the best lipid-related phenotypes in a 2^*nd*^-order analysis from univariate analysis of HDL using QMDR [5]. The second best model, rs10503669, has been reported to associate with LPL [16]. The third best model, rs2074356, associated with HDL [1]. All p-values selected by the multi-CMDR method were <10^−3^.

For 2^*nd*^-order analysis, the proposed multi-CMDR identified the best two SNP combinations, rs11216126 and rs4244457, where rs11216126 is reported to be related to HDL [17]. rs4244457 (LPL) occurs in the gene for the key enzyme responsible for the lipolytic processing of TG-rich lipoproteins [5]. Note that rs4244457 was selected as the most lipid-related SNP in a 1^*st*^- and 2^*nd*^-order analysis, using a multi-QMDR method for testing association with LDL [5]. Moreover, rs11600380, rs10503669, and rs16940212 were previously reported to relate to TG, LDL, and HDL, respectively [16, 18, 19]. Each of those three SNPs was also reported in previous studies, but as far as we know, there were no simultaneously reported 2^*nd*^-order interactions.

## 4. Discussion

For GGI analysis for multiple quantitative traits, we proposed multi-CMDR. Analyzing correlated multivariate phenotypes was shown to have higher power to detect susceptible genes and GGIs, by using more information from data [32]. The main feature differences between multi-QMDR and multi-CMDR lies in how to define groups for each combination cell. Multi-QMDR uses summary scores obtained by principal component analysis to classify high-risk and low-risk groups. The observations of each cell are assigned to the high-risk group if the local mean is greater than or equal to the global mean; otherwise the observations are assigned to the low-risk group. On the other hand, multi-CMDR divides groups using clustering. By comparing the global and local ratios, as calculated by using the membership degrees obtained through fuzzy k-means clustering, the observations of each cell are assigned to *D*_1_, if the local ratio is greater than or equal to the global ratio; otherwise the observations are assigned to *D*_2_.

This proposed multi-CMDR was shown to be less sensitive for outliers and nonsymmetric distributions than other methods. 10-fold cross-validation and Hotelling's *T*^2^ statistic were used to select the best model. In the simulation study, we showed that the proposed multi-CMDR could be used effectively in case of bivariate gamma distribution. While the proposed method did not seem to have advantage of computing time over the multi-QMDR method, it was higher for the skewed distribution. In real-life data analysis, multi-CMDR detected the best SNPs and 2-way interactions for lipid-related traits (HDL, TG, and LDL). The best SNPs, selected by our method, have been reported to associate with similar traits [1, 5, 15–19]. While our proposed method performs well for nonsymmetric distributions, it would be always worth to try appropriate transformations to make nonsymmetric distributions symmetric.

In terms of computation time efficiency, multi-QMDR was slightly faster than multi-CMDR. Using an AMD Ryzen 2700x desktop machine with 16G RAM, multi-QMDR took 145.8841 seconds on average (100 repetitions) to conduct real data analysis for the first-order interaction, whereas multi-CMDR took 162.7906 seconds on average. For simulation dataset with 400 sample size and 20 SNPs, multi-QMDR took 17.3334 seconds on average to conduct the 2^nd^-order interaction, while multi-CMDR took 19.3947 seconds on average. That is, when the number of SNPs is small, the difference in computation time is small. R program to conduct multi-CMDR is available at our github repository (https://github.com/stat17-hb/Multi-CMDR).

## 5. Conclusion

For the analysis of GGIs associated with multiple quantitative traits, we proposed a new extension of the MDR algorithm that includes clustering. Using fuzzy k-means clustering, we divided samples into two groups and trimmed outliers in noise cluster. By fuzzy k-means clustering, we can capture numerous attributes of multivariate data. Therefore, this is a very productive way to use values calculated from clusters to set thresholds to assign observations to specific groups, in that the proposed multi-CMDR uses a fuzzy k-means clustering method. Unlike k-means clustering, where each observation is assigned to only one cluster, fuzzy k-means clustering provides each observation with a degree of membership to each cluster. Fuzzy k-means clustering is especially useful when the cluster boundary is not clear, and it also allows outliers to be clustered into a noise cluster and reflects individual membership degrees of elements in the same cluster. We expect that multi-CMDR would improve the identification of gene-gene interactions associated with numerous multifactorial human pathologies.

## Figures and Tables

**Pseudocode 1:**
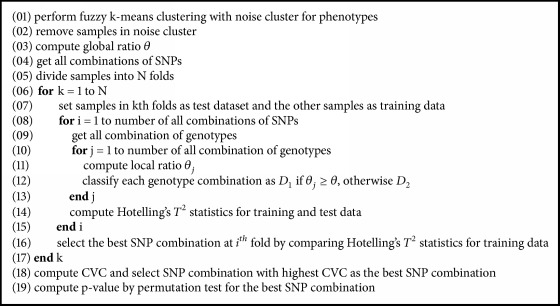
Pseudocode of multi-CMDR.

**Table 1:** Best models from 1^*st*^- and 2^*nd*^-order interaction analysis. *T*^2^ statistics were calculated from the test set.

Order	rs ID	Chr.	CVC	Hotelling's *T*^2^	p-value	Ref.
1^**st**^	rs11066280	12	4	2.86	<0.001	[5, 15]
	rs10503669	8	4	2.79	<0.001	[16]
	rs2074356	12	2	2.82	<0.001	[1]

2^**nd**^	rs11216126, rs4244457	11, 8	4	3.86	<0.001	[5, 17]
	rs11600380, rs10503669	11, 8	3	3.54	<0.001	[16, 18]
	rs11216126, rs10503669	11, 8	1	3.29	<0.001	[17, 18]
	rs16940212, rs10503669	15, 8	1	3.57	<0.001	[18, 19]
	rs16940212, rs4244457	15, 8	1	2.78	<0.001	[5, 19]

## Data Availability

The Korea Association Resource (KARE) project data will be publicly distributed by the Distribution Desk of Korea Biobank Network (https://koreabiobank.re.kr/). The data request should be made directly to Distribution Desk of Korea Biobank Network. Any inquiries should be sent to admin@koreabiobank.re.kr.

## Supplementary Materials

Supplementary Materials There were 10 combinations of minor allele frequencies (MAFs) and 5 models for each simulation setup. The MAF was 0.2, up to model 5. From models 6 to 10, the MAF was 0.4. We also considered correlation values of 0, 0.25, and 0.5. CMDR (multi-CMDR), MCMDR2 (multi-CMDR without trimming), MCMDR3 (multi-CMDR without membership score), MQMDR (multi-QMDR), QMDR. Y1 (QMDR with *Y*_1_), and QMDR Y2 (QMDR with *Y*_2_).
