# Supplementary material for: Multivariate Cluster-Based Multifactor Dimensionality Reduction to Identify Genetic Interactions for Multiple Quantitative Phenotypes
Source: Biomed Res Int. 2019 Jul 11;2019:4578983. doi: 10.1155/2019/4578983 (PMC6657635; doi:10.1155/2019/4578983)
Supplement: Supplementary Materials — There were 10 combinations of minor allele frequencies (MAFs) and 5 models for each simulation setup. The MAF was 0.2, up to model 5. From models 6 to 10, the MAF was 0.4. We also considered correlation values of 0, 0.25, and 0.5. CMDR (multi-CMDR), MCMDR2 (multi-CMDR without trimming), MCMDR3 (multi-CMDR without membership score), MQMDR (multi-QMDR), QMDR. Y1 (QMDR with Y1), and QMDR Y2 (QMDR with Y2). [file 4578983.f1.zip › 4578983_SupplDesc.docx]

There were 10 combinations of minor allele frequencies (MAFs), and 5 models for each simulation setup. The MAF was 0.2, up to model 5. From models 6 to 10, the MAF was 0.4. We also considered correlation values of 0, 0.25, and 0.5. CMDR (multi-CMDR), MCMDR2 (multi-CMDR without trimming, MCMDR3 (multi-CMDR without membership score), MQMDR (multi-QMDR), QMDR. Y1 (QMDR with 𝑌_1_), and QMDR Y2 (QMDR with 𝑌_2_)
