# Supplementary material for: Multivariate Cluster-Based Multifactor Dimensionality Reduction to Identify Genetic Interactions for Multiple Quantitative Phenotypes
Source: Biomed Res Int. 2019 Jul 11;2019:4578983. doi: 10.1155/2019/4578983 (PMC6657635; doi:10.1155/2019/4578983)
Supplement: Supplementary Materials — There were 10 combinations of minor allele frequencies (MAFs) and 5 models for each simulation setup. The MAF was 0.2, up to model 5. From models 6 to 10, the MAF was 0.4. We also considered correlation values of 0, 0.25, and 0.5. CMDR (multi-CMDR), MCMDR2 (multi-CMDR without trimming), MCMDR3 (multi-CMDR without membership score), MQMDR (multi-QMDR), QMDR. Y1 (QMDR with Y1), and QMDR Y2 (QMDR with Y2). [file 4578983.f1.zip › mat.4578983.v2.pdf]

Supplemental materials

There were 10 combinations of minor allele frequencies (MAFs), and 5 models for each simulation setup. The MAF was 0.2, up to model 5. From models 6 to 10, the MAF was 0.4. We also considered correlation values of 0, 0.25, and 0.5. CMDR (multi-CMDR), MCMDR2 (multi-CMDR without trimming, MCMDR3 (multi-CMDR without membership score), MQMDR (multi-QMDR), QMDR. Y1 (QMDR with Y_1), and QMDR Y2 (QMDR with Y_2)

Multivariate Normal Distributions

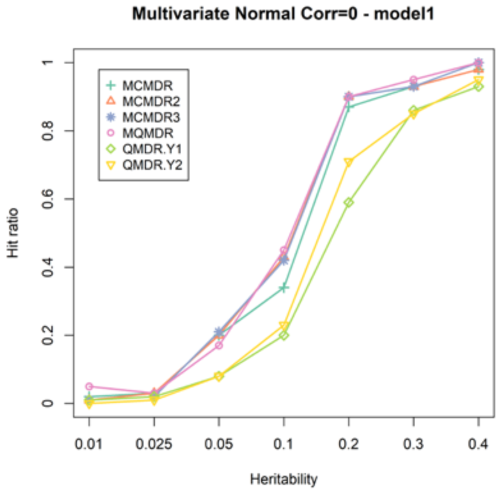

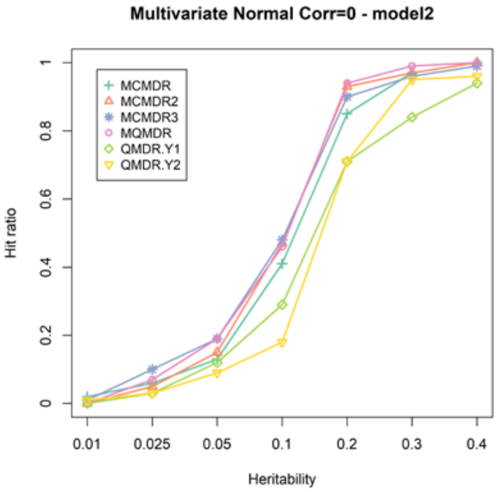

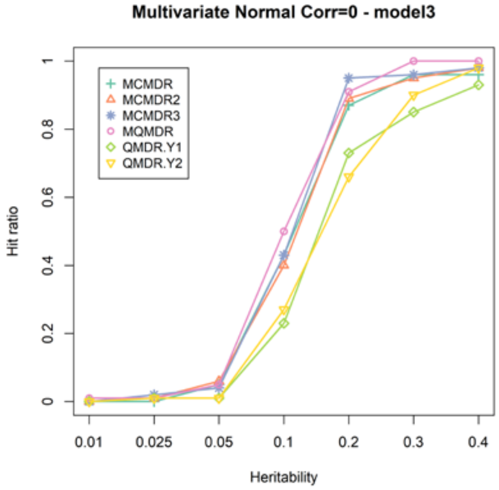

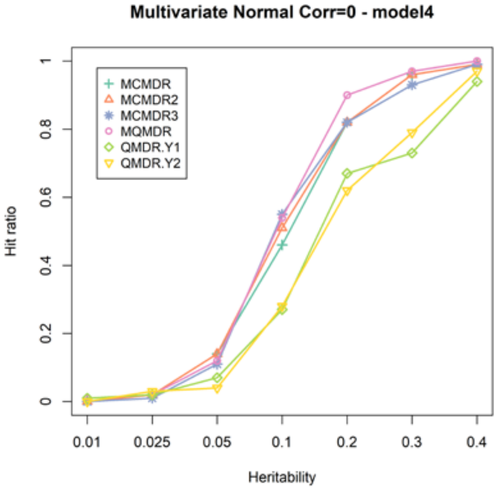

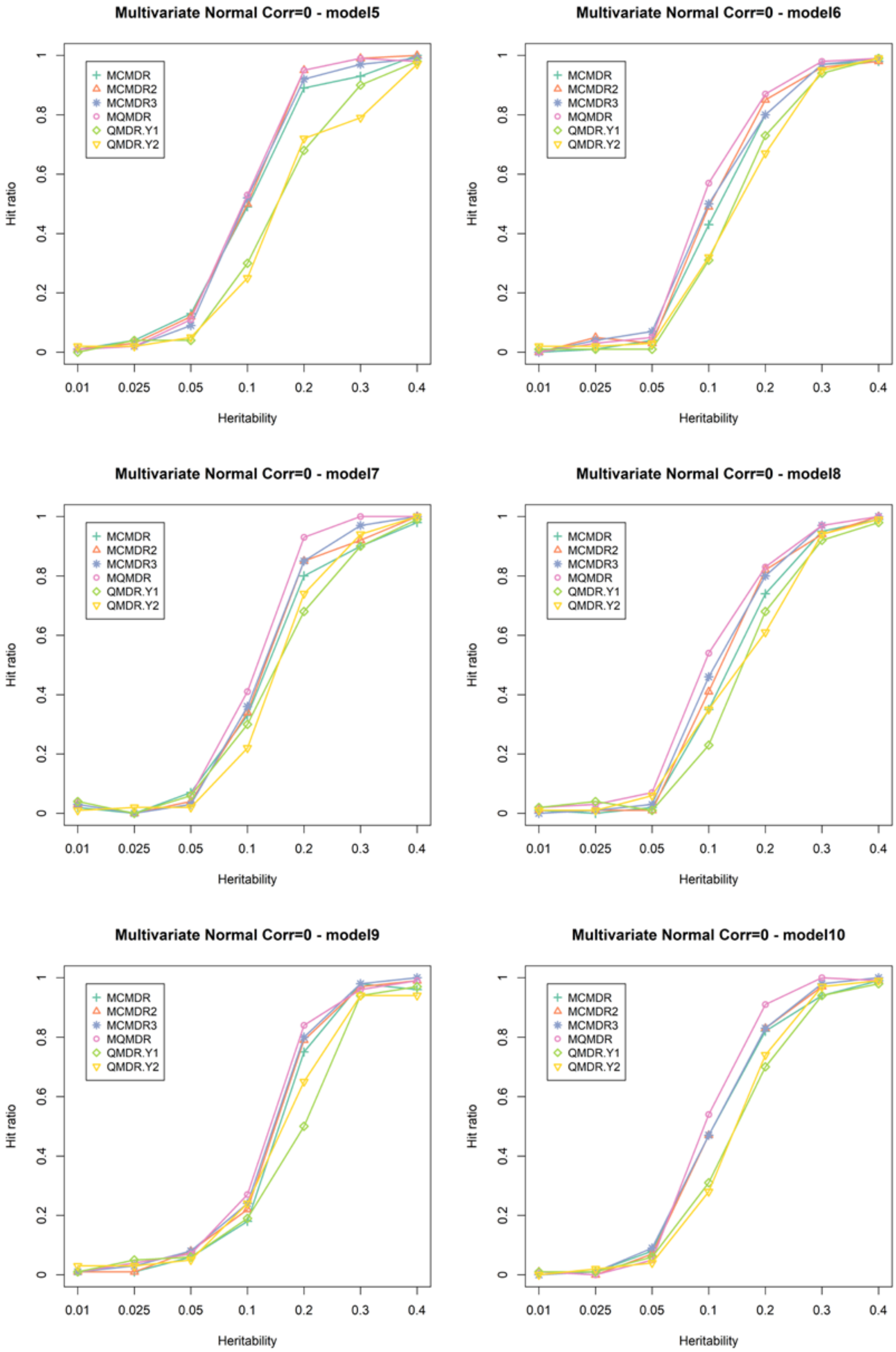

Supplemental Figure 1. Hit-ratios for multivariate normal distributions with zero correlation, for 10 models.

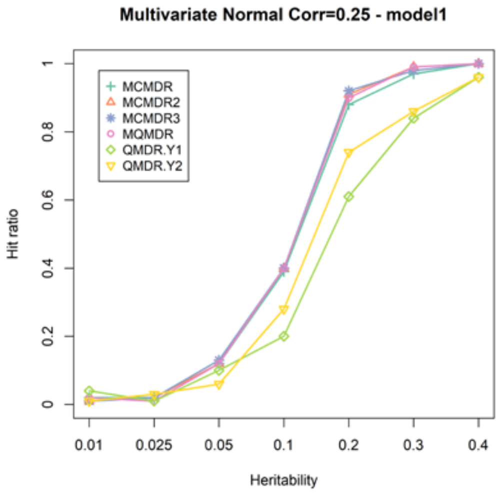
Multivariate Normal Corr=0.25 - model1
MCMDR
MCMDR2
MCMDR3
MQMDR
QMDR.Y1
QMDR.Y2
Hit ratio
Heritability

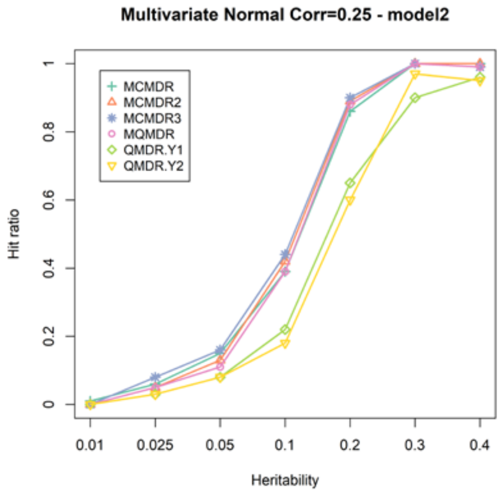
Multivariate Normal Corr=0.25 - model2
MCMDR
MCMDR2
MCMDR3
MQMDR
QMDR.Y1
QMDR.Y2
Hit ratio
Heritability

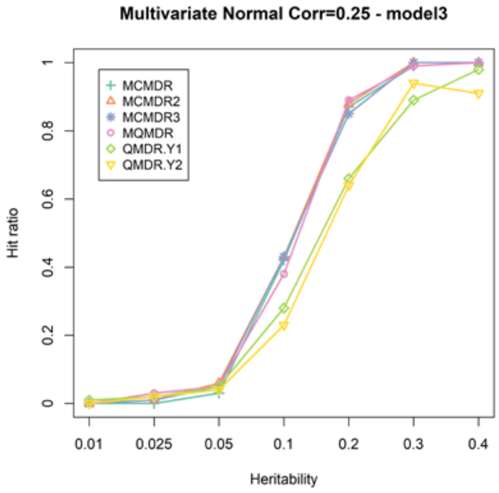
Multivariate Normal Corr=0.25 - model3
MCMDR
MCMDR2
MCMDR3
MQMDR
QMDR.Y1
QMDR.Y2
Hit ratio
Heritability

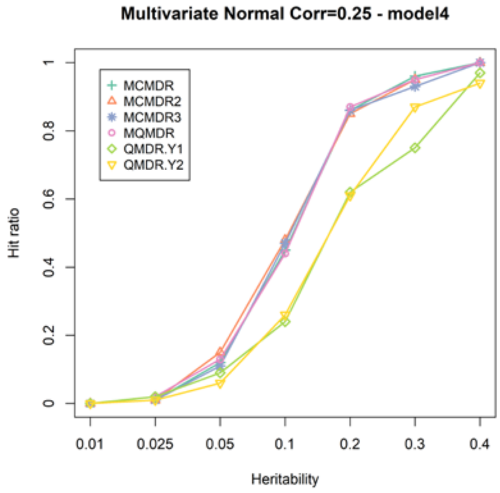
Multivariate Normal Corr=0.25 - model4
MCMDR
MCMDR2
MCMDR3
MQMDR
QMDR.Y1
QMDR.Y2
Hit ratio
Heritability

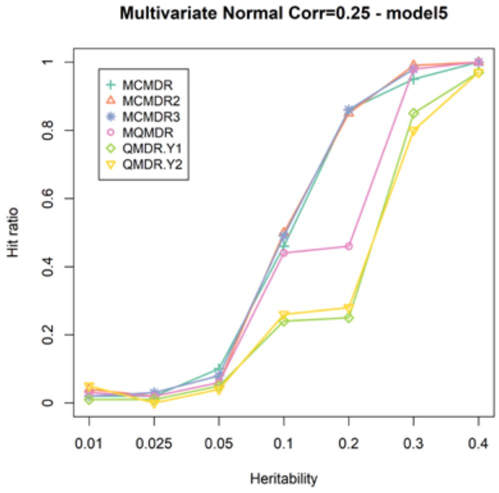
Multivariate Normal Corr=0.25 - model5
MCMDR
MCMDR2
MCMDR3
MQMDR
QMDR.Y1
QMDR.Y2
Hit ratio
Heritability

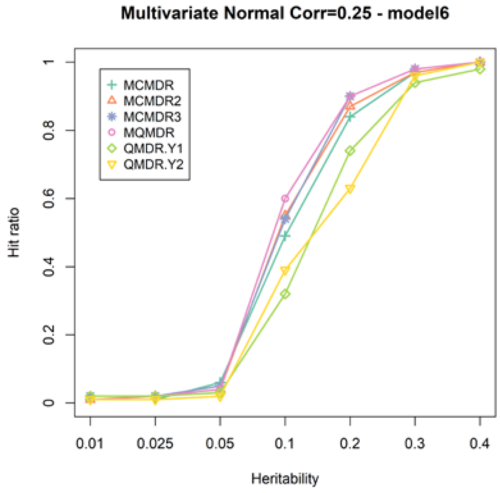
Multivariate Normal Corr=0.25 - model6
MCMDR
MCMDR2
MCMDR3
MQMDR
QMDR.Y1
QMDR.Y2
Hit ratio
Heritability

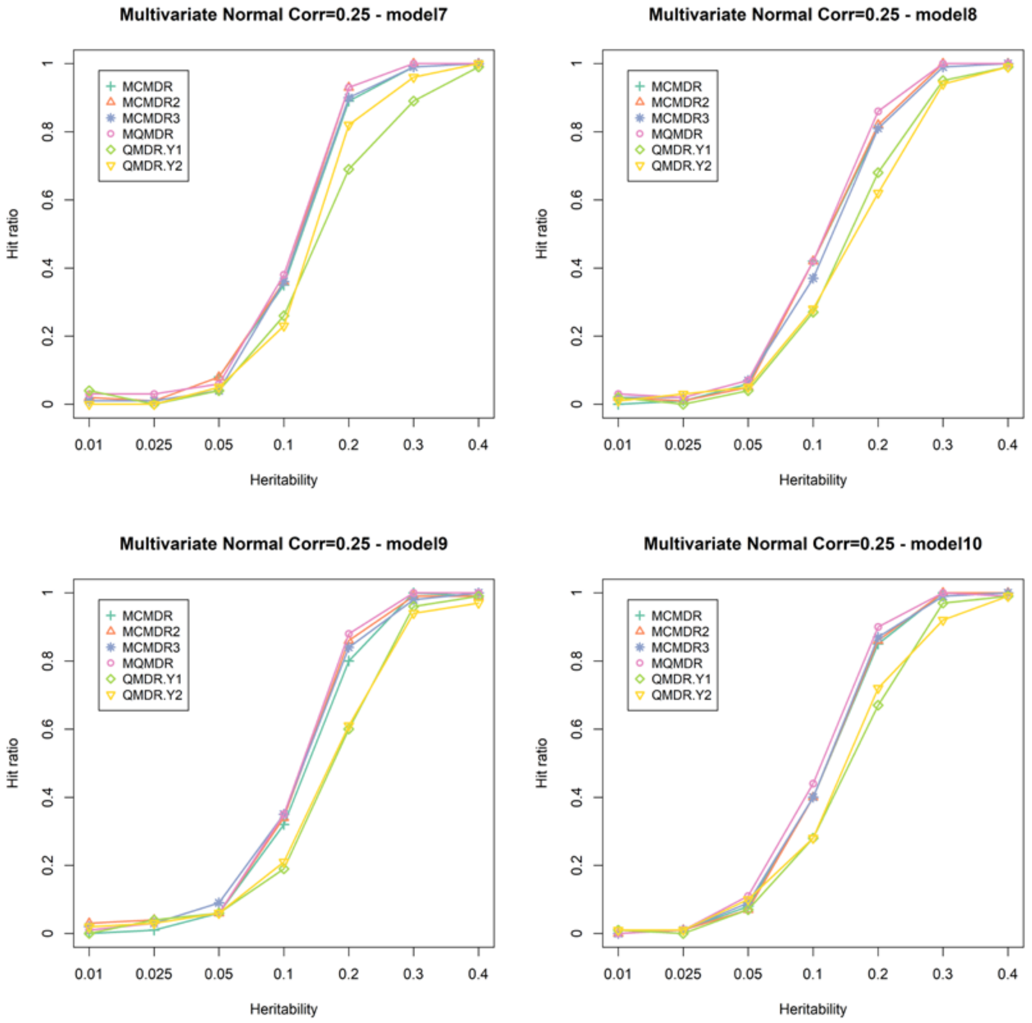

Supplemental Figure 2. Hit-ratios for multivariate normal distributions with correlation 0.25, for 10 models.

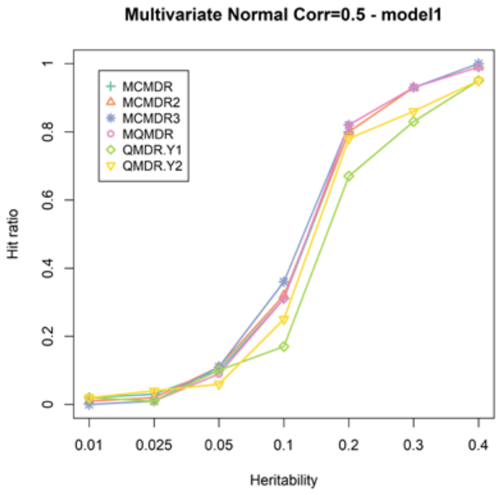
Multivariate Normal Corr=0.5 - model1
MCMDR
MCMDR2
MCMDR3
MQMDR
QMDR.Y1
QMDR.Y2
Hit ratio
0
0.2
0.4
0.6
0.8
1
0.01
0.025
0.05
0.1
0.2
0.3
0.4
Heritability

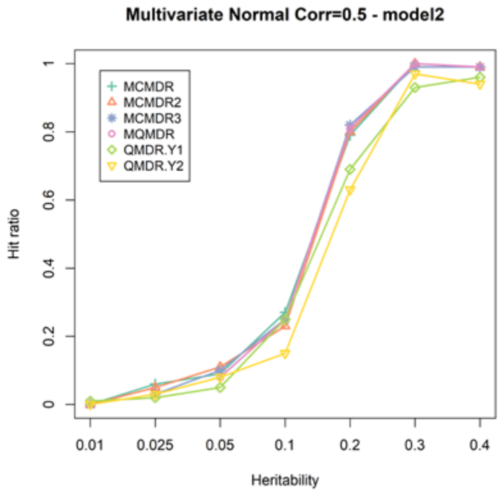
Multivariate Normal Corr=0.5 - model2
MCMDR
MCMDR2
MCMDR3
MQMDR
QMDR.Y1
QMDR.Y2
Hit ratio
0
0.2
0.4
0.6
0.8
1
0.01
0.025
0.05
0.1
0.2
0.3
0.4
Heritability

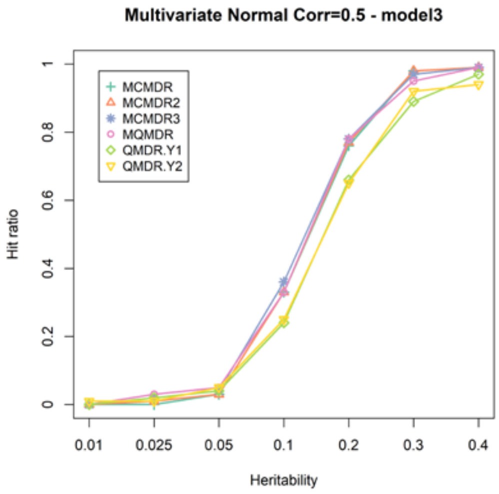
Multivariate Normal Corr=0.5 - model3
MCMDR
MCMDR2
MCMDR3
MQMDR
QMDR.Y1
QMDR.Y2
Hit ratio
0
0.2
0.4
0.6
0.8
1
0.01
0.025
0.05
0.1
0.2
0.3
0.4
Heritability

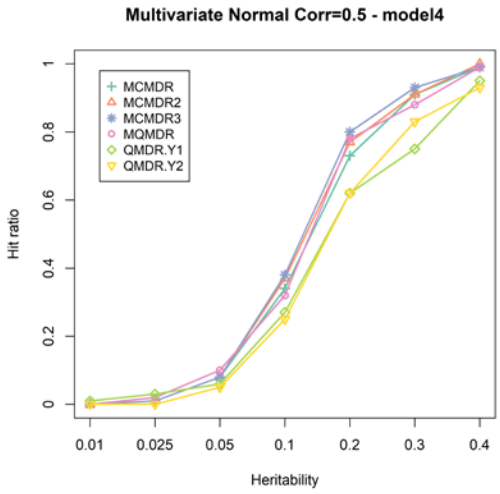
Multivariate Normal Corr=0.5 - model4
MCMDR
MCMDR2
MCMDR3
MQMDR
QMDR.Y1
QMDR.Y2
Hit ratio
0
0.2
0.4
0.6
0.8
1
0.01
0.025
0.05
0.1
0.2
0.3
0.4
Heritability

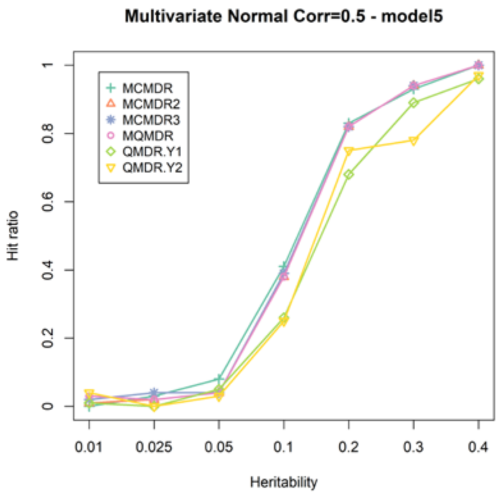
Multivariate Normal Corr=0.5 - model5
MCMDR
MCMDR2
MCMDR3
MQMDR
QMDR.Y1
QMDR.Y2
Hit ratio
0
0.2
0.4
0.6
0.8
1
0.01
0.025
0.05
0.1
0.2
0.3
0.4
Heritability

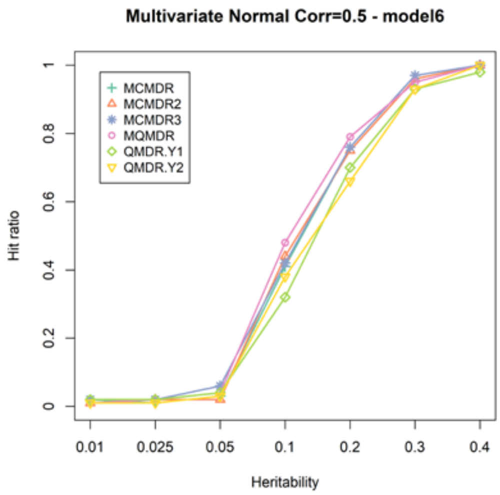
Multivariate Normal Corr=0.5 - model6
MCMDR
MCMDR2
MCMDR3
MQMDR
QMDR.Y1
QMDR.Y2
Hit ratio
0
0.2
0.4
0.6
0.8
1
0.01
0.025
0.05
0.1
0.2
0.3
0.4
Heritability

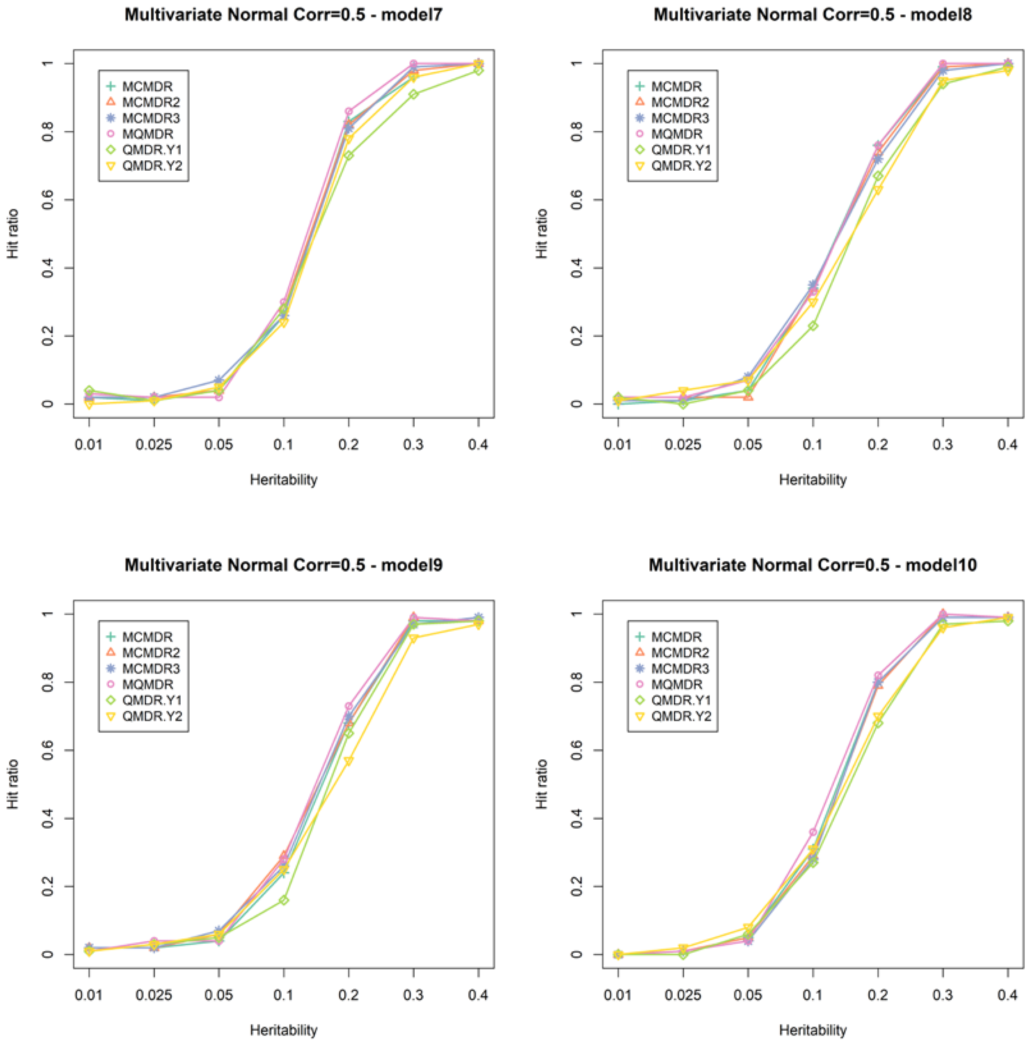

Supplemental Figure 3. Hit-ratios for multivariate normal distribution with correlation 0.5 for 10 models

Multivariate Gamma Distribution

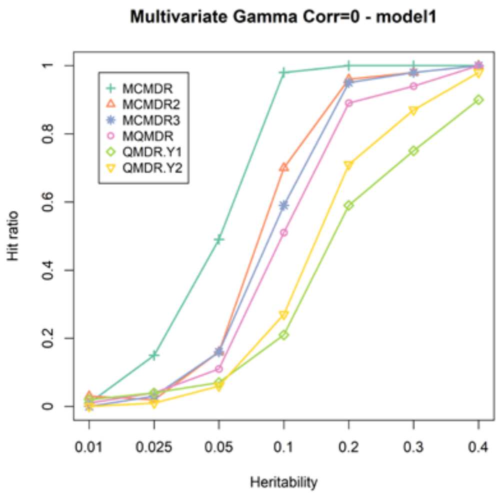

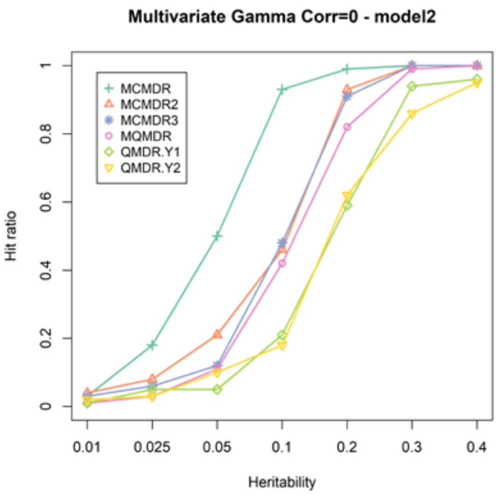

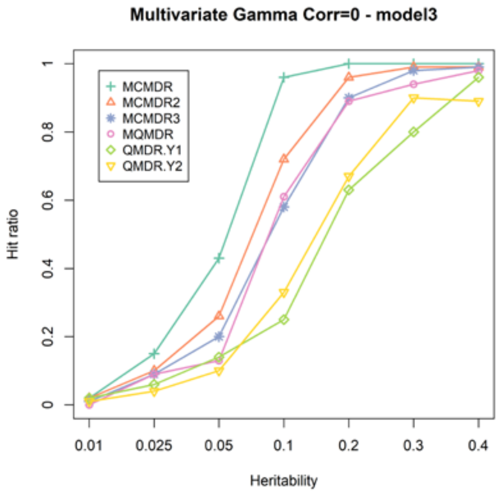

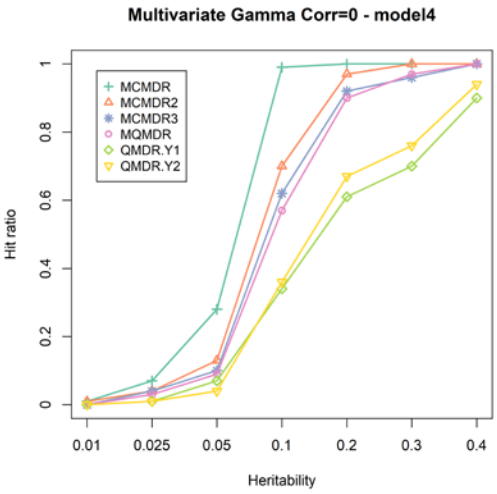

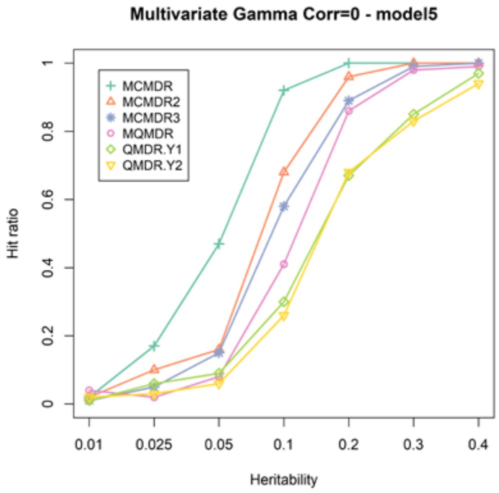

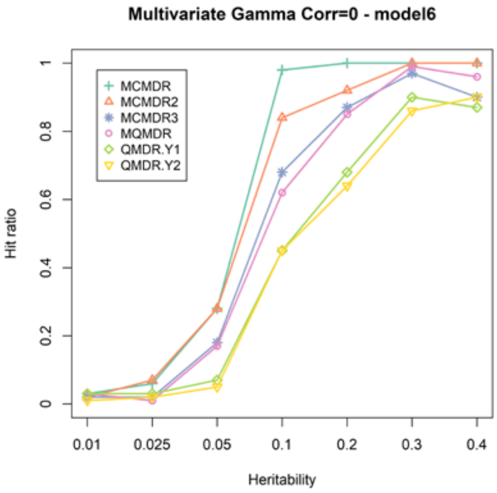

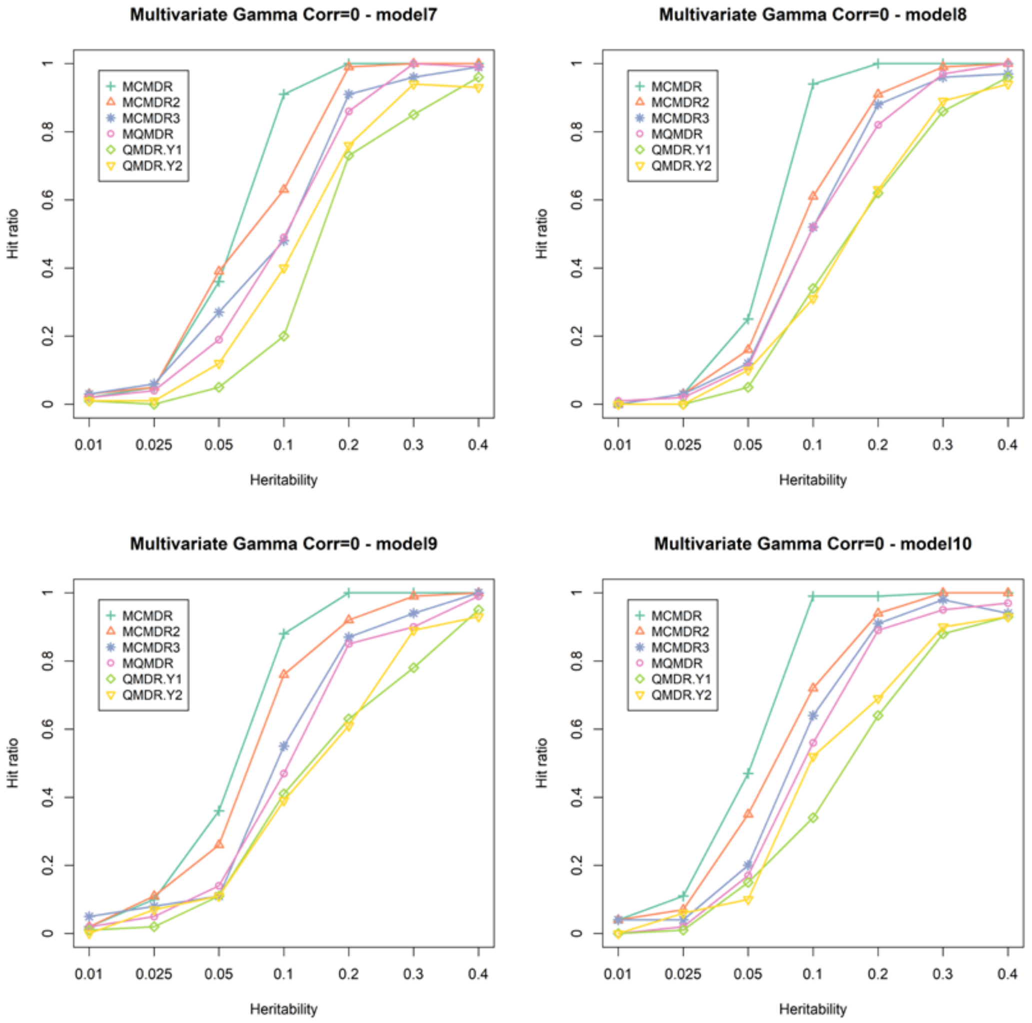

Supplemental Figure 4. Hit-ratios for multivariate gamma distributions, with zero correlation, for 10 models.

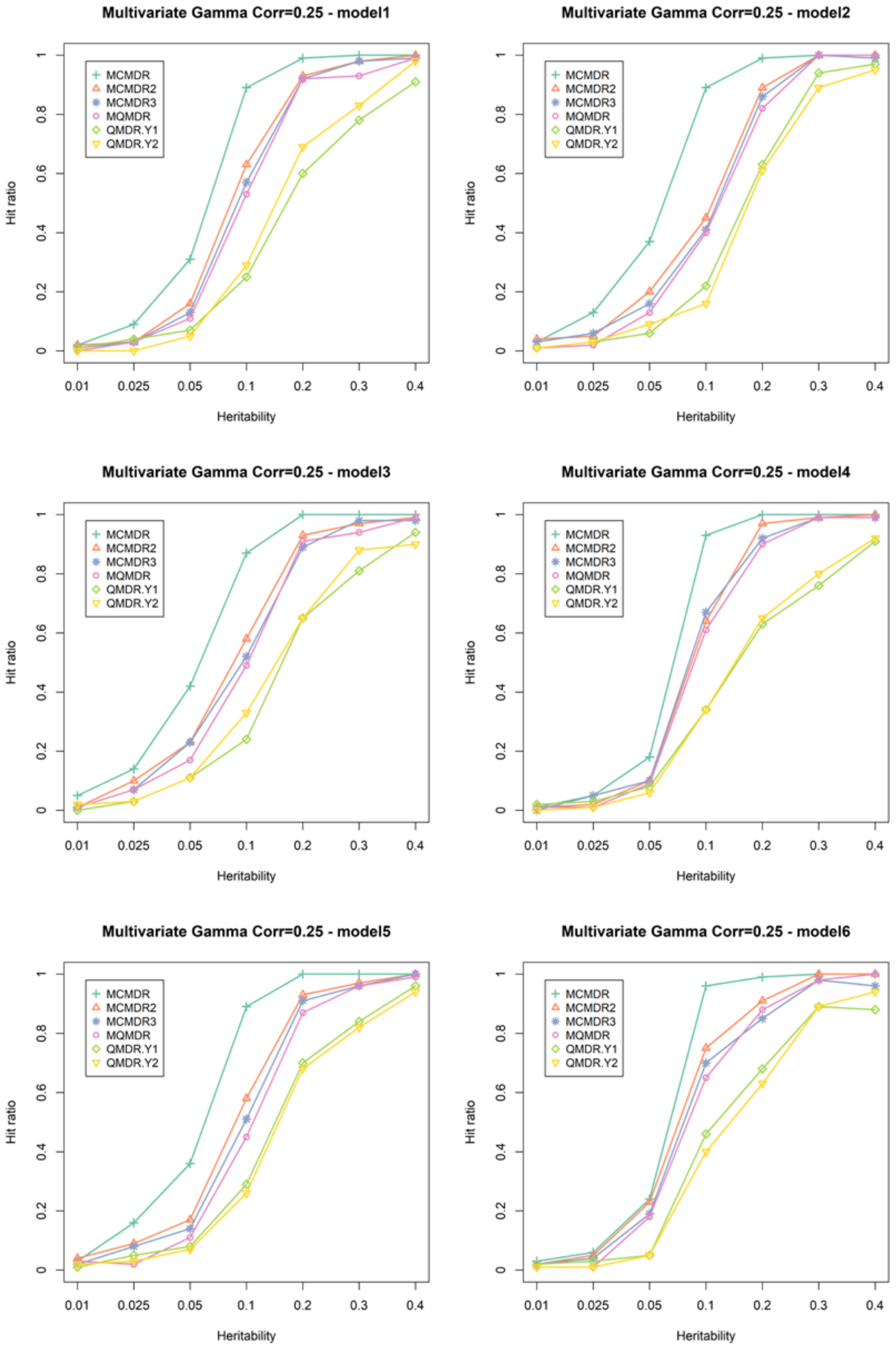
Multivariate Gamma Corr=0.25 - model1
Multivariate Gamma Corr=0.25 - model2
Multivariate Gamma Corr=0.25 - model3
Multivariate Gamma Corr=0.25 - model4
Multivariate Gamma Corr=0.25 - model5
Multivariate Gamma Corr=0.25 - model6
MCMDR
MCMDR2
MCMDR3
MQMDR
QMDR.Y1
QMDR.Y2
Hit ratio
0
0.2
0.4
0.6
0.8
1
0.01
0.025
0.05
0.1
0.2
0.3
0.4
Heritability

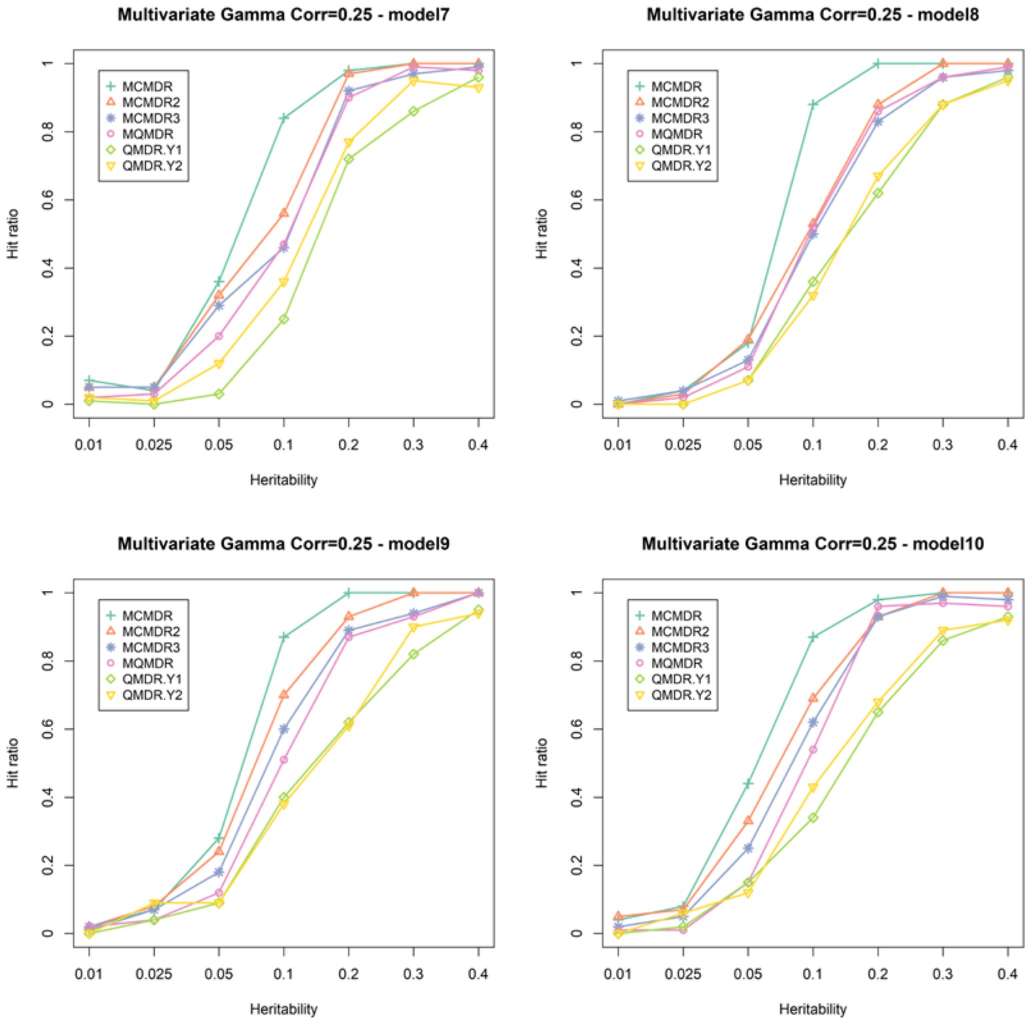

Supplemental Figure 5. Hit-ratios for multivariate gamma distributions, with correlation 0.25, for 10 models.

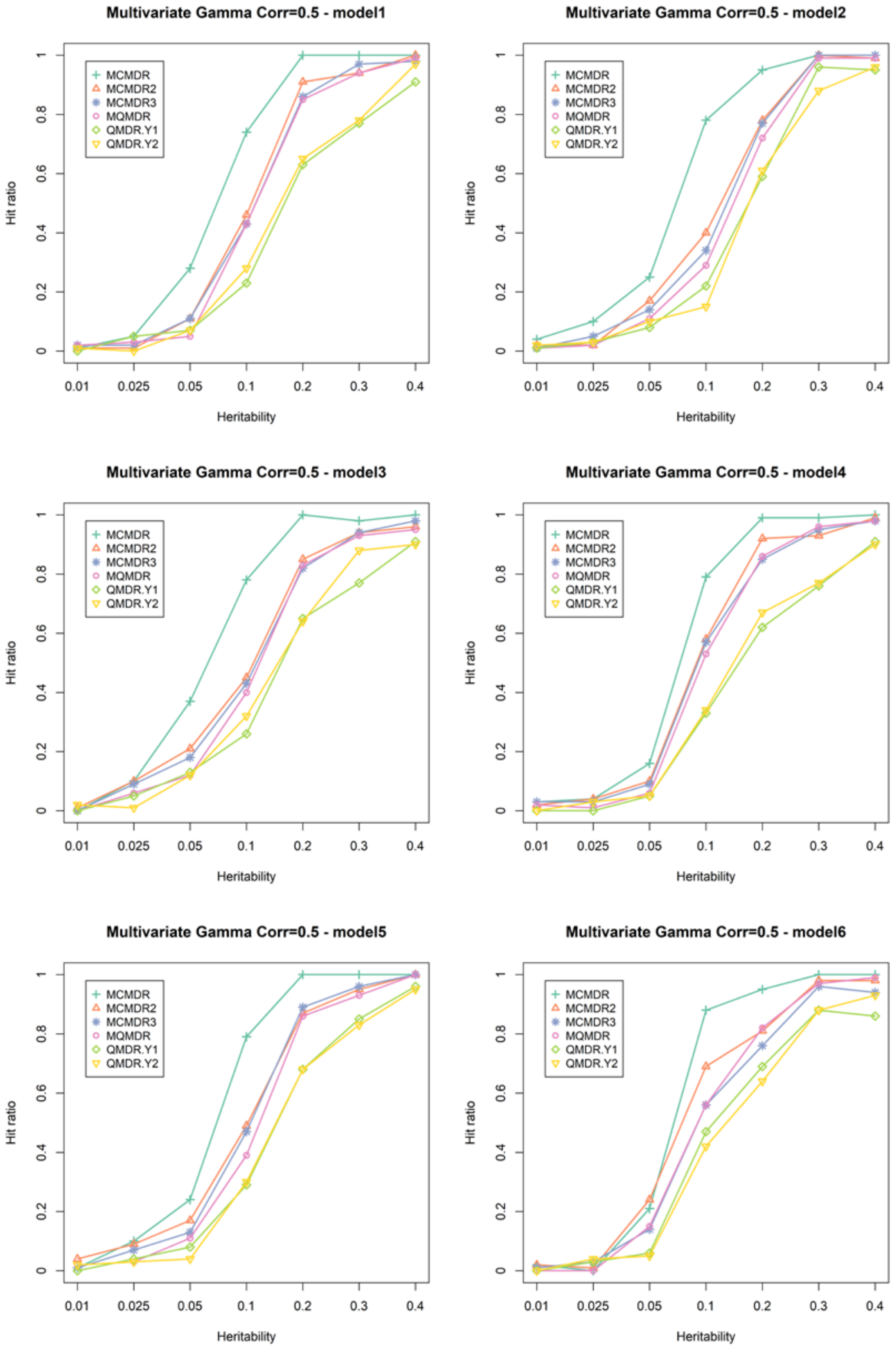
Multivariate Gamma Corr=0.5 - model1
Multivariate Gamma Corr=0.5 - model2
Multivariate Gamma Corr=0.5 - model3
Multivariate Gamma Corr=0.5 - model4
Multivariate Gamma Corr=0.5 - model5
Multivariate Gamma Corr=0.5 - model6
MCMDR
MCMDR2
MCMDR3
MQMDR
QMDR.Y1
QMDR.Y2
Hit ratio
0
0.2
0.4
0.6
0.8
1
0.01
0.025
0.05
0.1
0.2
0.3
0.4
Heritability

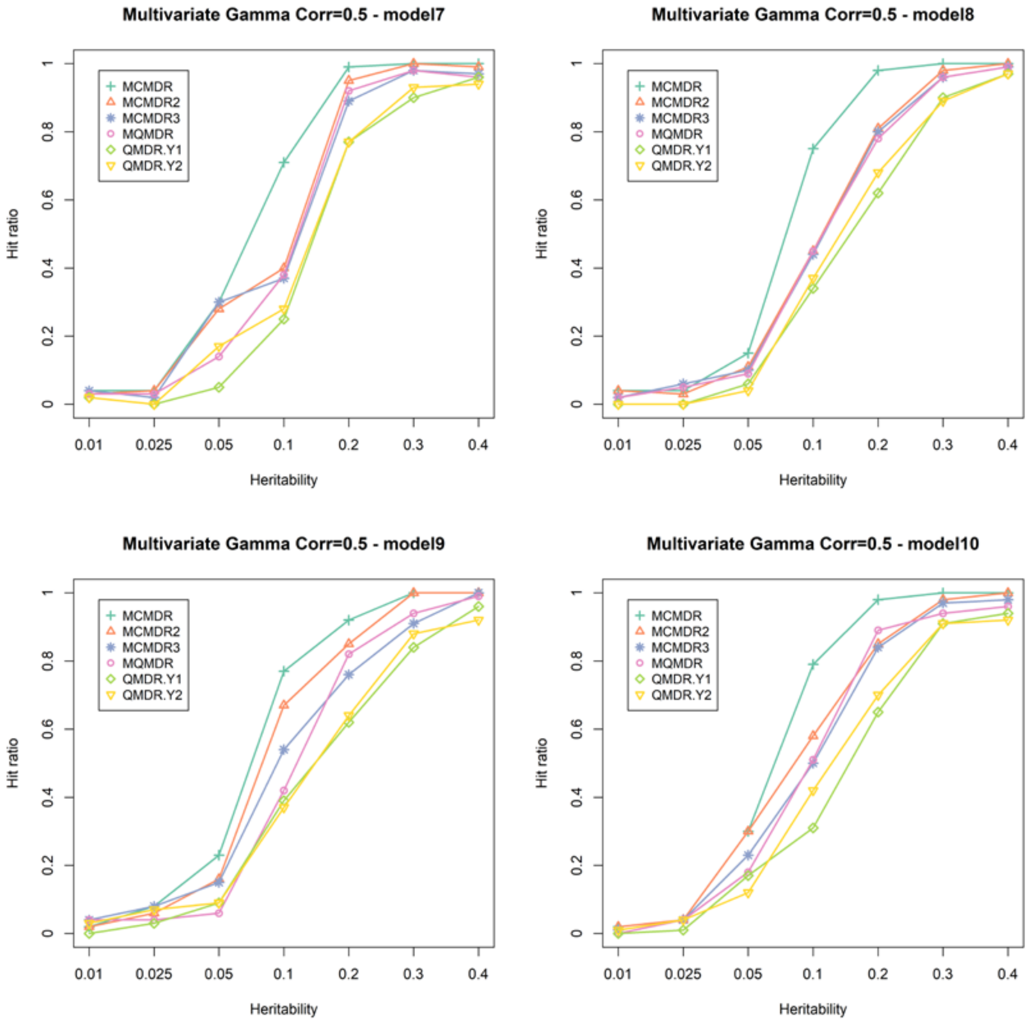

Supplemental Figure 6. Hit-ratios for multivariate gamma distributions, with correlation 0.5, for 10 models.

Multivariate normal distribution

	MCMDR1	MCMDR2	MCMDR3	MQMDR	QMDR.Y1	QMDR.Y2
**HET:0.01**	0.005	0.003	0.007	0.004	0.005	0.005
**HET:0.025**	0.005	0.007	0.006	0.005	0.007	0.004
**HET:0.05**	0.005	0.007	0.006	0.008	0.01	0.012
**HET:0.1**	0.004	0.002	0.002	0.004	0.005	0.005
**HET:0.2**	0.005	0.004	0.005	0.006	0.004	0.011
**HET:0.3**	0.009	0.005	0.005	0.007	0.003	0.007
**HET:0.4**	0.005	0.008	0.003	0.005	0.008	0.006

Supplemental Table 1. Empirical false positive rate for multivariate normal distributions with zero correlation

	MCMDR1	MCMDR2	MCMDR3	MQMDR	QMDR.Y1	QMDR.Y2
**HET:0.01**	0.006	0.001	0.004	0.006	0.003	0.006
**HET:0.025**	0.003	0.002	0.008	0.004	0.007	0.006
**HET:0.05**	0.008	0.011	0.012	0.006	0.008	0.009
**HET:0.1**	0.005	0.007	0.007	0.003	0.003	0.008
**HET:0.2**	0.005	0.004	0.004	0.008	0.005	0.005
**HET:0.3**	0.006	0.008	0.006	0.006	0.003	0.005
**HET:0.4**	0.004	0.005	0.01	0.006	0.005	0.005

Supplemental Table 2. Empirical false positive rate for multivariate normal distributions with correlation 0.25

	MCMDR1	MCMDR2	MCMDR3	MQMDR	QMDR.Y1	QMDR.Y2
**HET:0.01**	0.006	0.003	0.003	0.002	0.003	0.003
**HET:0.025**	0.003	0.001	0.006	0.003	0.007	0.004
**HET:0.05**	0.01	0.01	0.012	0.009	0.006	0.005
**HET:0.1**	0.003	0.004	0.003	0.003	0.004	0.005
**HET:0.2**	0.006	0.003	0.006	0.008	0.006	0.006
**HET:0.3**	0.007	0.008	0.006	0.007	0.006	0.005
**HET:0.4**	0.005	0.004	0.005	0.006	0.004	0.002

Supplemental Table 3. Empirical false positive rate for multivariate normal distributions with correlation 0.5

Multivariate gamma distribution

	MCMDR1	MCMDR2	MCMDR3	MQMDR	QMDR.Y1	QMDR.Y2
**HET:0.01**	0.007	0.003	0.008	0.003	0.005	0.001
**HET:0.025**	0.004	0.005	0.003	0.005	0.008	0.003
**HET:0.05**	0.008	0.007	0.005	0.008	0.006	0.008
**HET:0.1**	0.004	0.005	0.003	0.007	0.009	0.002
**HET:0.2**	0.006	0.006	0.003	0.004	0.008	0.003
**HET:0.3**	0.002	0.001	0.001	0.002	0.001	0.001
**HET:0.4**	0.002	0.003	0.003	0.004	0.005	0.009

Supplemental Table 4. Empirical false positive rate for multivariate gamma distributions with zero correlation

	MCMDR1	MCMDR2	MCMDR3	MQMDR	QMDR.Y1	QMDR.Y2
**HET:0.01**	0.005	0.004	0.003	0.006	0.005	0.002
**HET:0.025**	0.002	0.005	0.002	0.003	0.009	0.002
**HET:0.05**	0.004	0.007	0.008	0.007	0.007	0.004
**HET:0.1**	0.006	0.002	0.002	0.008	0.007	0.006
**HET:0.2**	0.004	0.006	0.003	0.003	0.01	0.002
**HET:0.3**	0.001	0.001	0.005	0.002	0.002	0
**HET:0.4**	0.002	0.003	0.008	0.004	0.006	0.006

Supplemental Table 5. Empirical false positive rate for multivariate gamma distributions with correlation 0.25

	MCMDR1	MCMDR2	MCMDR3	MQMDR	QMDR.Y1	QMDR.Y2
**HET:0.01**	0.004	0.003	0.003	0.005	0.001	0.007
**HET:0.025**	0.005	0.008	0.009	0.01	0.006	0.005
**HET:0.05**	0.004	0.004	0.007	0.004	0.007	0.004
**HET:0.1**	0.009	0.006	0.004	0.006	0.006	0.005
**HET:0.2**	0.005	0.005	0.003	0.004	0.01	0.004
**HET:0.3**	0.001	0.002	0.005	0.001	0.005	0.002
**HET:0.4**	0.001	0.004	0.003	0.004	0.005	0.002

Supplemental Table 6. Empirical false positive rate for multivariate gamma distributions with correlation 0.5
